# Stepwise Splitting Growth and Pseudocapacitive Properties of Hierarchical Three-Dimensional Co_3_O_4_ Nanobooks

**DOI:** 10.3390/nano7040081

**Published:** 2017-04-10

**Authors:** Huilong Chen, Shuang Lu, Feilong Gong, Huanzhen Liu, Feng Li

**Affiliations:** 1 State Laboratory of Surface and Interface Science and Technology, Zhengzhou University of Light Industry, Zhengzhou 450002, China; xunlingzhaohui@163.com (H.C.); ls102511@163.com (S.L.); longfei617381884@163.com (F.G.); huanzhenliuasd@sohu.com (H.L.); 2 American Advanced Nanotechnology, Houston, TX 77459, USA

**Keywords:** Co_3_O_4_, nanobooks, pseudocapacitors, stepwise splitting, hierarchical materials

## Abstract

Three-dimensional hierarchical Co_3_O_4_ nanobooks have been synthesized successfully on a large scale by calcining orthorhombic Co(CO_3_)_0.5_(OH)·0.11H_2_O precursors with identical morphologies. Based on the influence of reaction time and urea concentration on the nanostructures of the precursors, a stepwise splitting growth mechanism can be proposed to understand the formation of the 3D nanobooks. The 3D Co_3_O_4_ nanobooks exhibit excellent pseudocapacitive performances with specific capacitances of 590, 539, 476, 453, and 421 F/g at current densities of 0.5, 1, 2, 4, and 8 A/g, respectively. The devices can retain ca. 97.4% of the original specific capacitances after undergoing charge–discharge cycle tests 1000 times continuously at 4 A/g.

## 1. Introduction

Cobalt cobaltite (Co_3_O_4_) is one of the most promising materials in designing advanced devices for storing energy, and its capacitive properties have been investigated extensively in the last decade [1,2,3,4,5,6,7,8,9,10,11,12,13,14,15,16,17,18,19,20,21,22,23,24,25,26,27,28,29]. It was found that 3D hierarchical Co_3_O_4_ nanoarchitectures exhibit excellent electrochemical performances due to their abilities of facilitating both fast electron transportation and ion diffusion [27,28,29]. Our group has concentrated on tailoring the microstructures of functional materials for improving their performances in storing energy [26,27,28,29]. Compared to single crystalline Co_3_O_4_ particles grown on and around multi-walled carbon nanotube (MWCNT) [26], for instance, porous and single crystalline Co_3_O_4_ beads show much better capacitive properties [29]. Three-dimensional nanoarchitectures constructed with 2D Co_3_O_4_ nanowalls exhibit outstanding performances in storing energy [27]. Based on a multistep splitting growth of 1D nanorods, Co_3_O_4_ twin-spheres with excellent pseudocapacitive properties can be produced on a large scale [28]. While we have expected that 2D nanosheets could also split in a manner similar to 1D nanorods behaving in the formation of twin-spheres with sea urchin like structures [28], to the best of our knowledge, there has to date been no report in the literature concerned with the synthesis and formation of 3D Co_3_O_4_ nanobooks.

Herein we endeavor to report on porous 3D Co_3_O_4_ nanobooks and their pseudocapacitive properties. One-dimensional Co(CO_3_)_0.5_(OH)·0.11H_2_O precursor nanorods can be initially generated in solvothermal reactions for their fast growth in the (100) direction. The 1D nanostructures then convert into 2D nanoplates with a prolonged reaction time. Finally, the 2D nanoplates split to form 3D hierarchical Co(CO_3_)_0.5_(OH)·0.11H_2_O nanoarchitectures with book-like structures. After annealing at 400 °C, 3D porous and hierarchical Co_3_O_4_ nanoarchitectures carrying the book-like morphologies of their precursors were produced successfully on a large scale. Based on the influence of reaction time and urea concentration on the nanostructures of the precursors, a stepwise splitting growth mechanism of 2D nanoplates can be proposed to understand the formation of the 3D hierarchical precursors. The pseudocapacitors fabricated with as-prepared materials exhibit excellent pseudocapacitive performances with specific capacitances of 590, 539, 476, 453, and 421 F/g at current densities of 0.5, 1, 2, 4, and 8 A/g, respectively. The devices can retain ca. 97.4% of the original specific capacitances after undergoing charge–discharge cycling 1000 times continuously at 4 A/g. The high specific capacitances, cycle stability, and the rate of the electrodes made with the Co_3_O_4_ nanobooks could be attributed to their special 3D nanostructures with nanosized pores.

## 2. Experimental

### 2.1. Chemicals and Characterization

All of the reagents were analytically pure and were purchased from Shanghai Chemical Industrial Co. Ltd. (Shanghai, China) and used without further purification. The morphologies of the as-prepared samples were investigated with field emission scanning electron microscopy (FESEM, JEOL JSM-7001F, 10 kV, Tokyo, Japan) and transmission electron microscopy (TEM, JEOL JEM-2100, 200 kV, Tokyo, Japan). The compositions of the materials were characterized with X-ray diffraction (XRD) analysis using a Bruker AXS D8 advance diffractometer with Cu Kα radiation (Bruker, Advanced D8, Karlsruhe, Germany). The N_2_ adsorption and desorption isotherm was obtained using a Belsorp-Mini adsorption apparatus (Bel Japan Inc., Osaka, Japan). The pore size distribution was determined by using the BJH method applied to the desorption branch of adsorption–desorption isotherms.

### 2.2. Synthesis of Co_3_O_4_ Nanobooks

In a typical preparation, cobalt nitrate hexahydrate (Co(NO_3_)_2_·6H_2_O, 0.7271 g, 2.5 mmol) was first dissolved in 40 mL of a mix solvent consisting of ethylene glycol (EG, 20 mL) and deionized water (20 mL) to form a homogeneous solution under continuously stirring. Then, urea (NH_2_CONH_2_) (0.48 g, 8 mmol) was added into the solution. After stirring for another 30 min, the obtained solution was transferred into a Teflon-lined autoclave (50 mL) and kept at 160 °C for 20 h. After the autoclave was cooled naturally to room temperature, red powders were collected by centrifugation, washed with distilled water and ethanol 5 times each, and dried in vacuum at 60 °C for 12 h. Finally, the samples were calcined in a muffle furnace at 400 °C for 2 h in air, and then cooled to room temperature. The black powders of final products were collected for characterizations and applications.

### 2.3. Electrochemical Measurements

For the electrochemical measurements, active materials (Co_3_O_4_, 80 wt %), conductive material (acetylene carbon black, ATB, 10 wt %) and binder (polytetrafluoroethylene, PTFE, 10 wt %) were first mixed together and ground for ca. 10 min to obtain a mixture, which was then coated onto the surfaces of nickel meshes (ca. 1 × 1 cm) and dried at 100 °C for 12 h subsequently. The meshes with electrode materials (1.5 mg/cm) were finally pressed under 10 MPa to obtain working electrodes. The electrolyte used in the system was KOH aqueous solution (6 mol/L). All electrochemical measurements were carried out in a three-electrode experimental setup. Platinum foil and saturated calomel electrode (SCE) were used as counter electrode and reference electrode, respectively. Cyclic voltammogram (CV) was measured with an electrochemical workstation (CHI 660D, CH Instruments Inc., Shanghai, China). Galvanostatic charge–discharge cycle tests were performed on a LAND Cell test system CT2001A (Wuhan, China). The specific capacitances of the supercapacitors can be evaluated from the charge–discharge tests together with the following equation (Equation (1)) [1]:

*C_m_* = *I*∆*t*/*m*∆*V* (1)

where *C_m_* is the specific capacitance of the capacitor (F/g), *I* is the current of the charge–discharge (A), and ∆*t* is the discharging time period in seconds for the potential change ∆*V*. The *m* is the loaded mass of active materials. All of the electrochemical measurements were carried out at room temperature.

## 3. Results and Discussion

**Figure 1:**
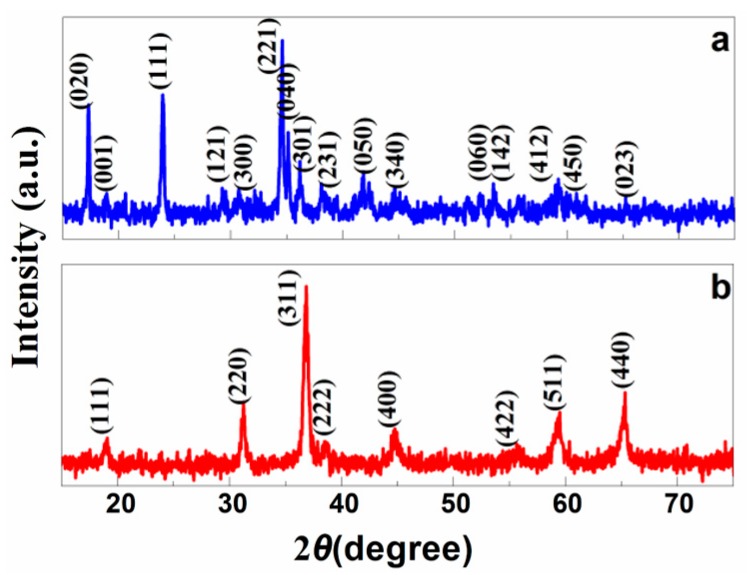
XRD profiles of (**a**) Co(OH)*_x_*(CO_3_)_0.5_·0.11H_2_O and (**b**) Co_3_O_4_.

The precursors and final products were first characterized with XRD as shown in Figure 1. All of the identified peaks in Figure 1a can be indexed to orthorhombic cobalt carbonate hydroxide hydrate-Co(CO_3_)_0.5_(OH)·0.11H_2_O (JCPDS Card No. 48-0083, *a* = 0.886 nm, *b* = 1.012 nm and *c* = 0.444 nm). After annealing at 400 °C for 2 h, the precursors discomposed completely and convert from orthorhombic Co(CO_3_)_0.5_(OH)·0.11H_2_O into cubic Co_3_O_4_, as shown in Figure 1b. The XRD profile (Figure 1b) of the final materials confirms that the heat-treated samples only contain cubic Co_3_O_4_phase (JCPDS Card No. 42-1467, *a* = 0.8037). The high intensities of the peaks indicate that the as-prepared materials are well crystallized. No peaks are attributed to impurity in the pattern (5% deviation), which confirms the formation of single-phase Co(CO_3_)_0.5_(OH)·0.11H_2_O and Co_3_O_4_.

**Figure 2:**
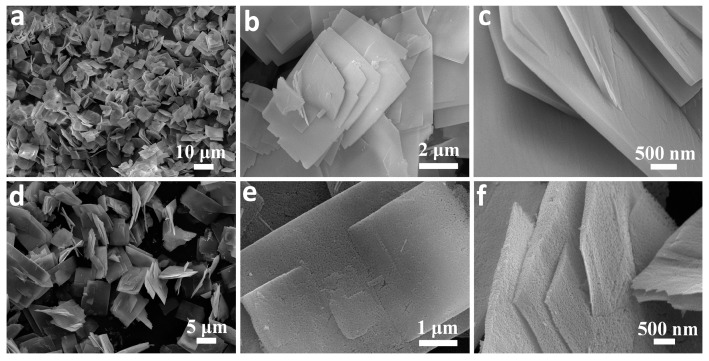
FESEM images of (**a**–**c**) Co(CO_3_)_0.5_(OH)·0.11H_2_O and (**d**–**f**) porous Co_3_O_4_ nanobooks.

Figure 2a–c shows the FESEM images of as-prepared orthorhombic Co(CO_3_)_0.5_(OH)·0.11H_2_O precursors. Uniform and well-dispersed 3D hierarchical nanostructures (Figure 2a) consisting of rectangular nanoplates of ca. 10 and 8 µm in length and width, respectively, have been produced successfully. Higher magnification SEM images (Figure 2b,c) of the materials further reveal that the particles are mainly composed of 2D nanoplates of 2–5 generations growing out from the central area of nanoplate substrates to form 3D nanoarchitectures with book-like structures. The nanoplates with thicknesses ranging from 50 to 100 nm have smooth surfaces. In contrast, while the final Co_3_O_4_ materials obtained maintain the 3D hierarchical book-like morphology of the precursors, many pores are generated throughout the materials (Figure 2e,f) after they are annealed at 400 °C for 2 h. The porous nanostructures can be attributed to the decomposition of Co(CO_3_)_0.5_(OH)·0.11H_2_O precursor and the release of H_2_O and CO_2_ from the system.

**Figure 3:**
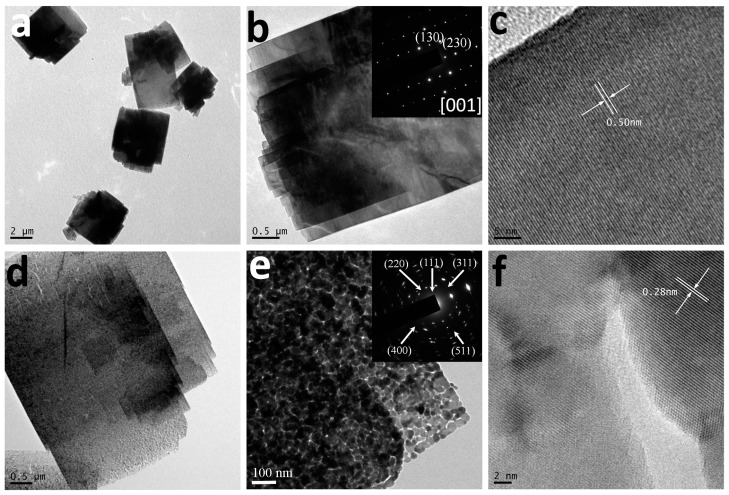
(**a**,**b**) TEM and (**c**) HRTEM images of single crystalline Co(CO_3_)_0.5_(OH)·0.11H_2_O nanobooks. (**d**,**e**) TEM and (**f**) HRTEM images of 3D hierarchical Co_3_O_4_ nanobooks. Insets in (**b**,**e**): the selective area electron diffraction (SAED) patterns of the precursor and final product.

More details concerned with the microstructures of the 3D precursor and porous Co_3_O_4_ nanobooks can be revealed by TEM and selective area electron diffraction (SAED). Figure 3a,b shows low magnification TEM images of several Co(CO_3_)_0.5_(OH)·0.11H_2_O nanobooks with smooth surfaces, which agrees well with the FESEM observations. The SAED pattern (insert in Figure 3b) composed of highly ordered dots, which can be attributed to the zone axis of (001) of orthorhombic Co(CO_3_)_0.5_(OH)·0.11H_2_O crystal phase, indicates that the nanoplates are single crystalline. The top and bottom planes of the nanoplates can therefore be indexed as the (001) plane of orthorhombic Co(CO_3_)_0.5_(OH)·0.11H_2_O. Figure 3c shows a high-resolution TEM (HRTEM) image recorded near the edge of a precursor nanoplate. The measured d-spacings of 0.50 nm correspond to the (020) plane of orthorhombic Co(CO_3_)_0.5_(OH)·0.11H_2_O. The results clearly verify that the orthorhombic Co(CO_3_)_0.5_(OH)·0.11H_2_O precursors grow in their (100) and (010) directions, respectively, to form rectangular nanoplates.

In contrast, the TEM image of the final Co_3_O_4_ nanobooks clearly shows porous structures across the particles. Many irregular pores from a few to tens of nanometers randomly distribute on the surface of the 3D Co_3_O_4_ nanobook as shown in Figure 3d,e. In addition, the TEM image as shown in Figure 3e reveals that the nanoplates consist of nanoparticles interconnected each other with a grain size of ca. 20 nm in diameter. The SAED pattern (inset in Figure 3e) of a Co_3_O_4_ nanoplate confirms the polycrystalline feature of the final products. The resolved fringes of 0.28 nm from the HRTEM image (Figure 3f) correspond to the (222) facet of cubic Co_3_O_4_.

**Figure 4:**
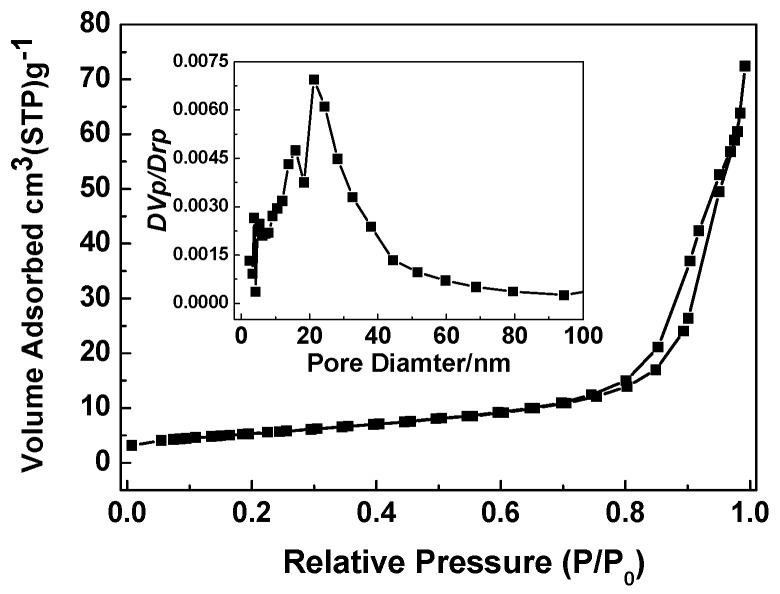
Typical nitrogen adsorption–desorption isotherm and pore-size distribution curve (inset) of Co_3_O_4_ nanobooks.

The specific surface area and pore volume of Co_3_O_4_ nanobooks were measured by using N_2_ adsorption and desorption experiments. Figure 4 shows the adsorption–desorption isotherm and the corresponding BJH pore size distribution plot (inset in Figure 4) of the materials. The existence of hysteresis loops between the isotherms indicates the adsorption–desorption characteristic of porous materials. The porous Co_3_O_4_ nanobooks with a total pore volume of 0.11 cm^3^/g have a surface area of 19.45 m^2^/g. Using the BJH method and desorption branch of nitrogen isotherm, we can calculate the size and shape of the pores, and the results indicate that the pores are not uniform in the Co_3_O_4_ nanobooks, and most of them are around 21.28 nm in diameter.

**Figure 5:**
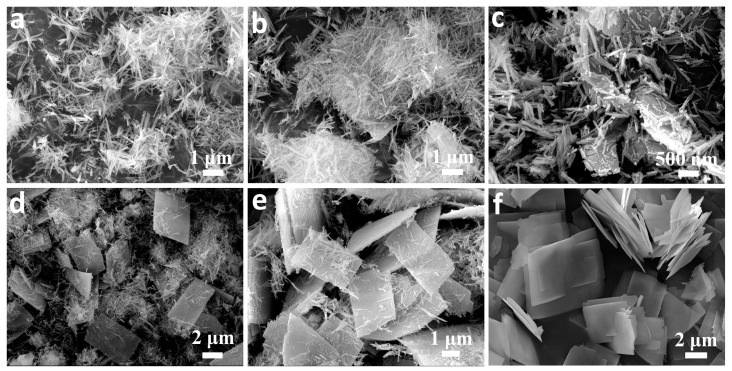
Morphology evolution of Co(CO_3_)_0.5_(OH)·0.11H_2_O precursors synthesized at 160 °C for (**a**) 2, (**b**) 3, (**c**) 4, (**d**) 5, (**e**) 6, and (**f**) 10 h, respectively.

In order to understand the formation of the 3D hierarchical nanobooks, the reaction time effect on the morphology of the Co(CO_3_)_0.5_(OH)·0.11H_2_O precursor was carefully investigated by keeping the other reaction parameters identical. The structural evolution of the precursors is presented in Figure 5. One-dimensional nanorods of ca. 2 µm in length and 20 nm in diameter (Figure 5a) were first produced after reacting for 2 h. After prolonging the reaction time to 3 h, nanoplates appeared in the products (Figure 5b). More 2D nanoplates as shown in Figure 5c formed in the mixtures after reacting for 4 h. Many rectangular nanoplates were generated after 5 h of reaction, but nanorods still existed in the materials (Figure 5d). After performing the reaction for 6 h, the population of nanorods further reduced, in contrast to the generation of more nanoplates with a single layer (Figure 5e). Three-dimensional hierarchical nanobooks consisting of rectangular nanoplates with smooth surfaces (Figure 5f) were finally produced on a large scale, and the nanorods disappeared almost completely after conducting the reaction for up to 10 h. Based on the reaction time effect on the morphology of the precursors, it can be concluded safely that 3D hierarchical Co(CO_3_)_0.5_(OH)·0.11H_2_O nanobooks form at the expense of 1D nanorods.

**Figure 6:**
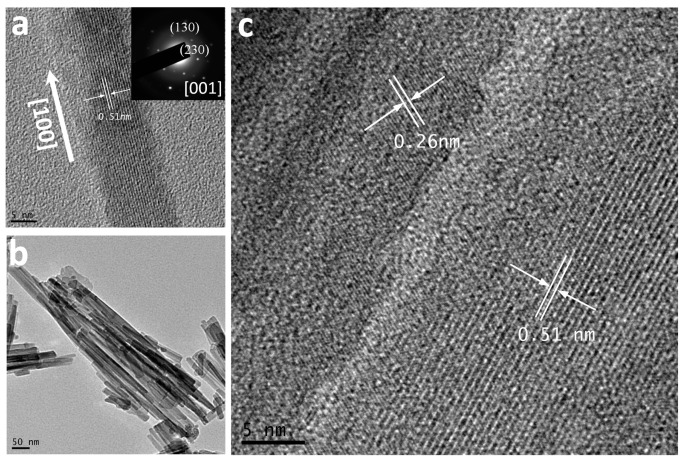
(**a**) HRTEM image of an individual Co(CO_3_)_0.5_(OH)·0.11H_2_O nanorod. (**b**) TEM image of Co(CO_3_)_0.5_(OH)·0.11H_2_O nanorods. (**c**) HRTEM image of two nanorods.

**Figure 7:**
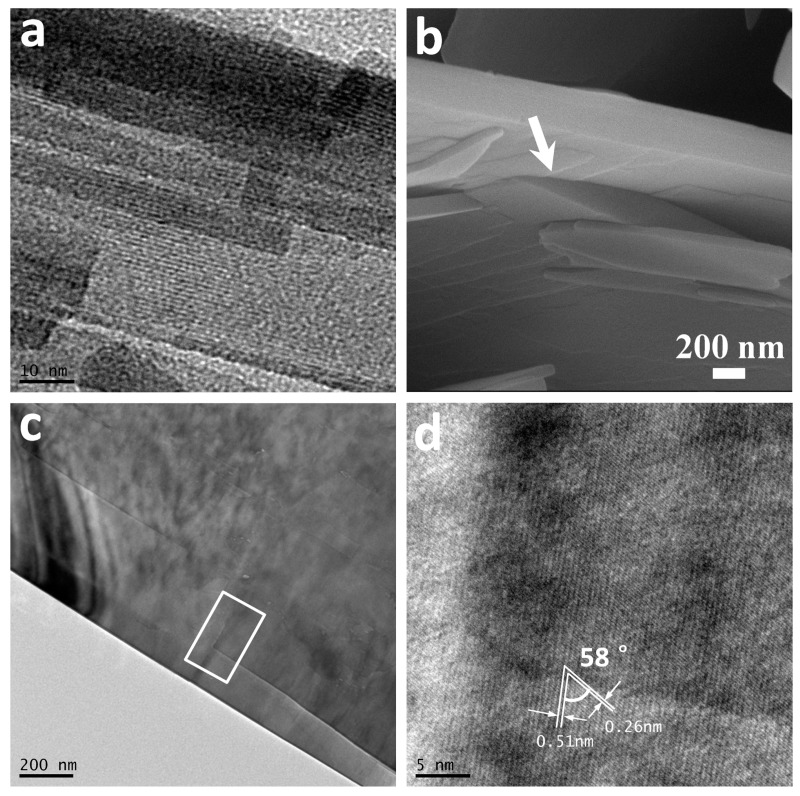
(**a**) TEM image of a Co(CO_3_)_0.5_(OH)·0.11H_2_O nanoplate with steps. (**b**–**d**) FESEM, TEM and HRTEM images at junction area of two nanoplates in a 3D hierarchical nanobook.

Figure 6a,b shows the TEM and HRTEM image of a Co(CO_3_)_0.5_(OH)·0.11H_2_O nanorod of ca. 10 nm in diameter prepared by reacting for 2 h. The inset in Figure 6a depletes the SAED pattern of a whole nanorod as shown in Figure 6a. The measured d-spacings as shown in Figure 6a,c are 0.51 nm and 0.26 nm, respectively, which corresponds to the (020) and (320) lattice fringe of orthorhombic Co(CO_3_)_0.5_(OH)·0.11H_2_O. These results indicate that Co(CO_3_)_0.5_(OH)·0.11H_2_O grow along its (100) axis to form single crystalline 1D nanorods. The orientations along the length, width, and thickness of the rectangular precursor can therefore be indexed to be (100), (010), and (001), respectively. The fast growth of Co(CO_3_)_0.5_(OH)·0.11H_2_O in their (100) directions can generate 1D nanorods. The nanorods then grow in their (010) directions and further convert to 2D rectangular nanoplates in consuming the nanorods smaller in dimension due to Ostwald ripening. During the formation of rectangular nanoplates, the nucleate and growth can also take place at the central areas of the nanoplates for the higher energy in the regions. Many steps can therefore be generated in the central area of the nanoplates as shown in Figure 7a. The white arrow in the FESEM image (Figure 7b) of a nanobook further highlights the steps in its central area. Two nanoplates have fused perfectly together to form a 3D hierarchical nanobook, and the corresponding fringes and intersection angle agree well with the orthorhombic Co(OH)*_x_*(CO_3_)_0.5_·0.11H_2_O.

**Figure 8:**
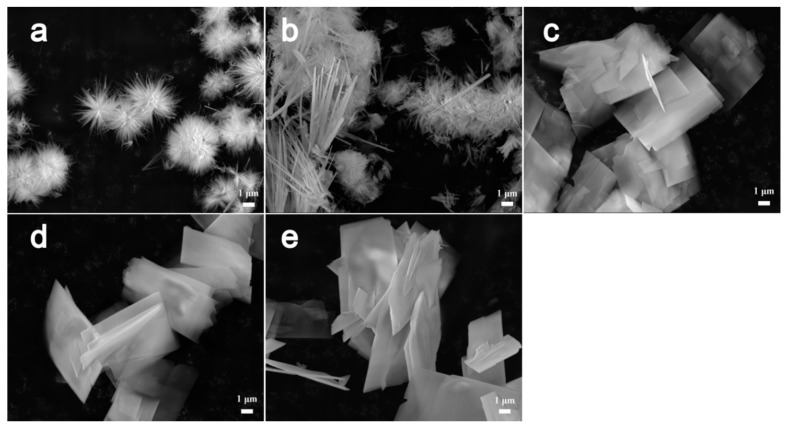
SEM images of precursors synthesized at 160 °C for 12 h by adding urea with (**a**) 1, (**b**) 3, (**c**) 6, (**d**) 10, and (**e**) 15 mmol intothe reactions, respectively.

The effect of the urea on the nanostructures of the precursors was also investigated carefully by adding different amounts of the reactant into the reaction and keeping the other reaction parameters identical. Figure 8 shows the structure evolution of the precursors accompanying the increase of urea amount added to the reactions. Three-dimensional hierarchical nanospheres consisting of nanowires can be initially produced by adding a small amount of urea tothe reaction (Figure 8a). The structures of the products finally change into 3D nanobooks (Figure 8c) composed of 2D nanosheets after adding urea with a concentration of more than 6 mmol into the system. It has been pointed out that the carbonate and hydroxyl anions generated by the hydrolysis of urea can directly affect crystal growth [2]. The reactions involved in our system could be described as follows (Equations (2)–(5)):

NH_2_CONH_2_ + H_2_O → NH_3_ + CO_2_ (2)

NH_3_ +H_2_O → NH_4_^+^ +OH^−^ (3)

CO_2_+ H_2_O → CO_3_^2−^ + 2H^+^ (4)

Co^2+^ + OH^−^ + CO_3_^2−^ + 0.11H_2_O → Co(CO_3_)_0.5_OH·0.11H_2_O (5)

The homogeneous formation of the precursors, which can be induced by the controlled generation of OH^−^ and CO_3_^2−^ ions for the slow hydrolysis rate of urea, shows an apparent advantage in the structure control of final products in comparison with conventional preparations of materials based on precipitation. Urea has therefore also played an important role in controlling the nanostructures of the precursors.

**Figure 9:**
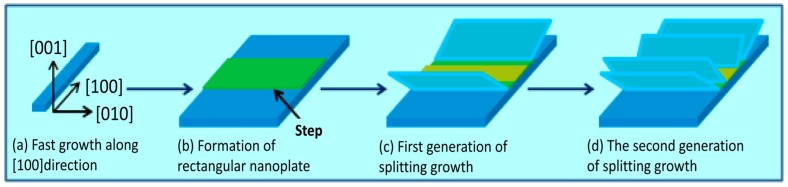
The multistep splitting growth of 3D hierarchical nanobook.

Based on the experimental observation, a stepwise splitting growth (Figure 9) of 2D nanoplates can be proposed to understand the formation of the 3D nanobooks. One-dimensional nanorods can be initially produced in the reactions because of the highest energy of the (100) facets of orthorhombic Co(CO_3_)_0.5_(OH)·0.11H_2_O and thus their fast growth in the (100) direction (Figure 9a). However, the energy required to grow the crystals into longer 1D nanorods also increases as the nanorods lengthen. Consequently, the energy for growing precursor nanorods in (010) directions could finally catch up with the energy required for growing in the (100) direction. The nanorods therefore convert into 2D nanoplates with rectangular shape for the growths in both the (100) and (010) directions at the same time (Figure 9b) at the expense of nanorods with smaller sizes due to Ostwald ripening. The continued nucleation and growths of nanocrystals prefer to take place at the steps and defects on their surfaces because of their higher energy in comparison to the smooth planes. The new generation of 2D nanoplates can therefore form at the central areas of the substrate nanoplates from the steps (Figure 9c). The stepwise growth of the nanoplates from the steps in the central areas of the nanoplate substrate eventually leads to the formation of 3D hierarchical materials with book-like structures (Figure 9d). It is still unclear, however, what has resulted in the 2D nanoplates orienting in directions that are different from those of the nanoplate substrates, while the perturbations could contribute to triggering the growth.

**Figure 10:**
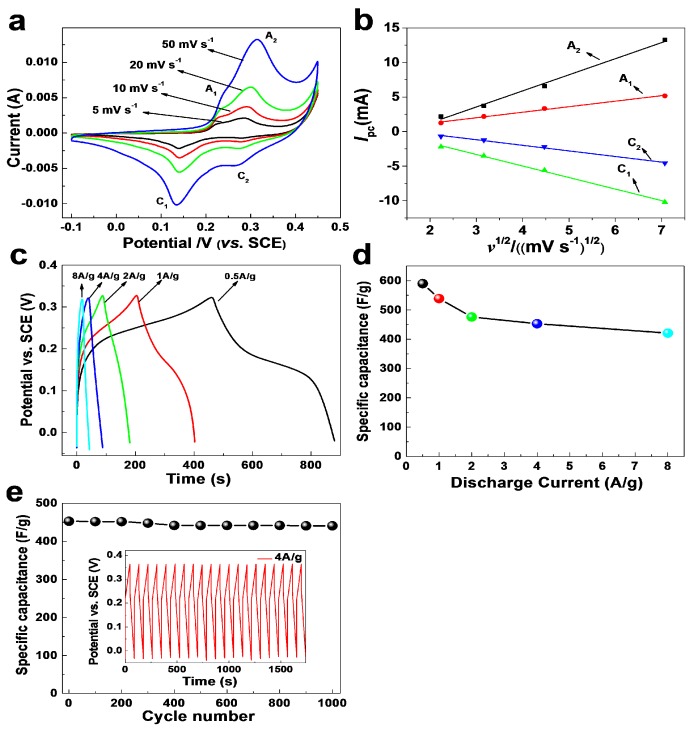
(**a**) CV of Co_3_O_4_ nanobooks in KOH electrolyte and (**b**) the relationship between the CV peak current (*I*_p_) and the square root of the scan rate (*v*). (**c**) Charge-discharge curves of porous Co_3_O_4_ nanoplates in KOH electrolyte (6 mol/L). (**d**) Average specific capacitances of the supercapacitors at different discharge current densities. (**e**) Average specific capacitances versus cycle number of the devices at galvanostatic charge and discharge current density of 4 A/g. The inset shows galvanostatic charge–discharge curves of the devices constructed with porous Co_3_O_4_ nanoplates at current density of 4 A/g.

Co_3_O_4_ materials are promising candidates for designing supercapacitors for their relative low environmental foot print, low cost, and specific capacitance of 3560 F/g in theory. We made electrodes with as-prepared 3D Co_3_O_4_ nanobooks for testing their performances in storing energy. The electrochemical properties of the materials were first studied by cyclic voltammetry (CV) in a KOH electrolyte (6 mol/L) as shown in Figure 10a. The shapes of the CV curves are distinctly different from the CV curves of electrochemical double layer capacitors. Two pair of redox peaks can be observed during the anodic and cathodic sweeps, which correspond to the conversion between different oxidation states of cobalt according to Equations (6) and (7). The first redox couple A_1_/C_1_ corresponds to the conversion between CoOOH and Co_3_O_4_, and the second redox couple A_2_/C_2_ is attributed to the change between CoOOH and CoO_2_. The plots of peak current density (*I*_p_) versus square root of sweep rate (*v*) for cathodic peaks (Figure 10b) follow well a power-law relationship with the sweep rate (*I*_p_ = *av*). A *b*-value of 0.5 indicates that the current is controlled by semi-infinite linear diffusion, and a *b*-value of 1 shows that the current is surface-controlled. The electrode kinetics under the conditions investigated is a diffusion-controlled battery-type Faradaic process. The CV curves demonstrate the characteristic charge storage of the pseudocapacitive process originating from reversible redox reactions. It can also be observed that the potentials of the anodic and cathodic peaks shift toward more anodic and cathodic directions, respectively, as scan rates increase, which indicates the quasi-reversible feature of the redox couples.

Co_3_O_4_ + OH^−^ + H_2_O ⇔ 3CoOOH + e (6)

CoOOH + OH^−^ ⇔ CoO_2_ + H_2_O + e. (7)

Figure 10c shows the galvanostatic charge–discharge curves of the supercapacitors made with porous Co_3_O_4_ nanobooks at different charge–discharge current densities with the potential window of −0.05–0.35 V. According to Equation (1), the discharge specific capacitance values (Figure 10d) of the porous Co_3_O_4_ nanobook electrodes calculated from the discharge curves are 590, 539, 476, 453, and 421 F/g at the current density of 0.5, 1, 2, 4, and 8 A/g, respectively. The decrease of the capacitances with the increase of the discharge current density is likely caused by the increase of the potential drop due to the resistance of the nanobooks and the relatively insufficient faradic redox reaction of active materials under higher discharge current densities. The specific capacitance value of the electrode retains ca. 71% and reaches 421 F/g at 8 A/g, compared to its value at 0.5 A/g. The excellent rate performances of porous Co_3_O_4_ nanobooks working at high current density are significant in practical applications of supercapacitors and could be attributed to the high efficiency of utilizing their active surface with nanosized pores and 3D hierarchical structures.

The cycle stability of supercapacitors is another crucial parameter for their practical applications. Figure 10e presents the cycling performances of the supercapacitors constructed with porous Co_3_O_4_ nanobooks under a current density of 4 A/g within the potential window of −0.05–0.35 V. A small decrease of specific capacitance can be observed after being cycled 300 times, which could be due to the consumption of electrode materials and binder for the redox taking place on the surface of the electrode. The capacitances of the devices remain almost constant after that, and they can retain approximately 97.4% of their maximum values after being cycled 1000 times. The results show the excellent stability of the electrodes assembled with 3D Co_3_O_4_ nanobooks. The galvanostatic curves (inset in Figure 10e) of the materials at the current density of 4 A/g show that the charge–discharge process of the electrode is highly reversible. Compared to the Co_3_O_4_ nanowires [7], hexagonal platelets [11], porous nanoplates [22], monolayer hollow spheres [12], nanorod assembled nanospheres [15], and porous particles [16] reported in the literature, as-prepared nanobooks show much higher specific capacitance at a high current density of 8 A/g. Our Co_3_O_4_ nanobooks also show comparable specific capacitance to hollow fluffy cages [1], porous nanostructures [14], twin-spheres [28], and hierarchical nanoarchitectures [27]. The materials also exhibit much higher specific capacitances in comparison with MoS_2_ [30] and α-Fe_2_O_3_ [31], and they are also comparable to CoS_2_ [32] and NiCo_2_S_4_ [33]. We could further improve the electrochemical performances of our nanobooks through constructing hybrids with carbon materials or transitional metal sulfides [34]. It was found that the microstructures, including their sizes and pores, affect their electrochemical performances dramatically, and we can also tailor the structures of the materials by adjusting the reaction parameters, thus improving their capabilities in storing energy. 

## 4. Conclusions

In summary, we have successfully demonstrated an approach for the scalable synthesis of porous 3D hierarchical Co_3_O_4_ nanobooks, based on a stepwise splitting growth of 2D nanoplates. The supercapacitors made with the materials show excellent electrochemical performances in rate and stability, which could be attributed to the porous 3D hierarchical structure of the nanobooks. By simply adjusting the reaction parameters, one can tailor the microstructures of the electrode materials and tune their electrochemical properties effectively. The stepwise splitting growth of 2D nanoplates could open a new pathway for constructing 3D hierarchical materials storing energy.
